# Supplementary material for: In-hospital areas with distinct maintenance and staff/patient traffic have specific microbiome profiles, functions, and resistomes
Source: mSystems. 2024 Jul 9;9(8):e00726-24. doi: 10.1128/msystems.00726-24 (PMC11334533; doi:10.1128/msystems.00726-24)

Supplementary Material

Table S1: Sampling locations and sites sorted by confinement level (CL1 - operating room (highly restricted); CL2 - patient-related areas (restricted); and CL3 - non-patient-related areas (not or only partially restricted).

Department	Sampled Surface	Confinement
**ICU non-patient-related areas:**		Level 3 (CL3)
	Floor	
**Toilet**	Sink	
	Toilet flush button	
**Waiting area**	Floor*	
	Door handle	
	Floor*	
**Nurses´ station**	Work desk	
	Sink	
**ICU patient-related areas:**		Level 2 (CL2)
	Floor*	
**2 bed patient room**	Bedframe	
	Area around touchscreen	
	Medical cart	
	Floor*	
**Examination room**	Touchscreen & pump station	
	Medical cart	
	Bedframe	
	Floor	
**Air lock**	Sink	
	Door & door handle	
	Floor*	
**Isolation room**	Touchscreen & pump station	
	Medical cart	
	Bedframe	
**Operating room:**		Level 1 (CL1)
	Floor*	
	Area around keyboard	
	Medical cart	
	Touchscreen Monitoring system	
	Vacuum machine	
	Surgical light	
	Keyboard	
	Door release button	
	Swiveling system	
	Remote control & surface	

Fig. S1: Sampling sites of ICU department (A) and operating room (B). Samples collected are marked with x (CL3 - yellow; CL2 - orange; CL1- red).

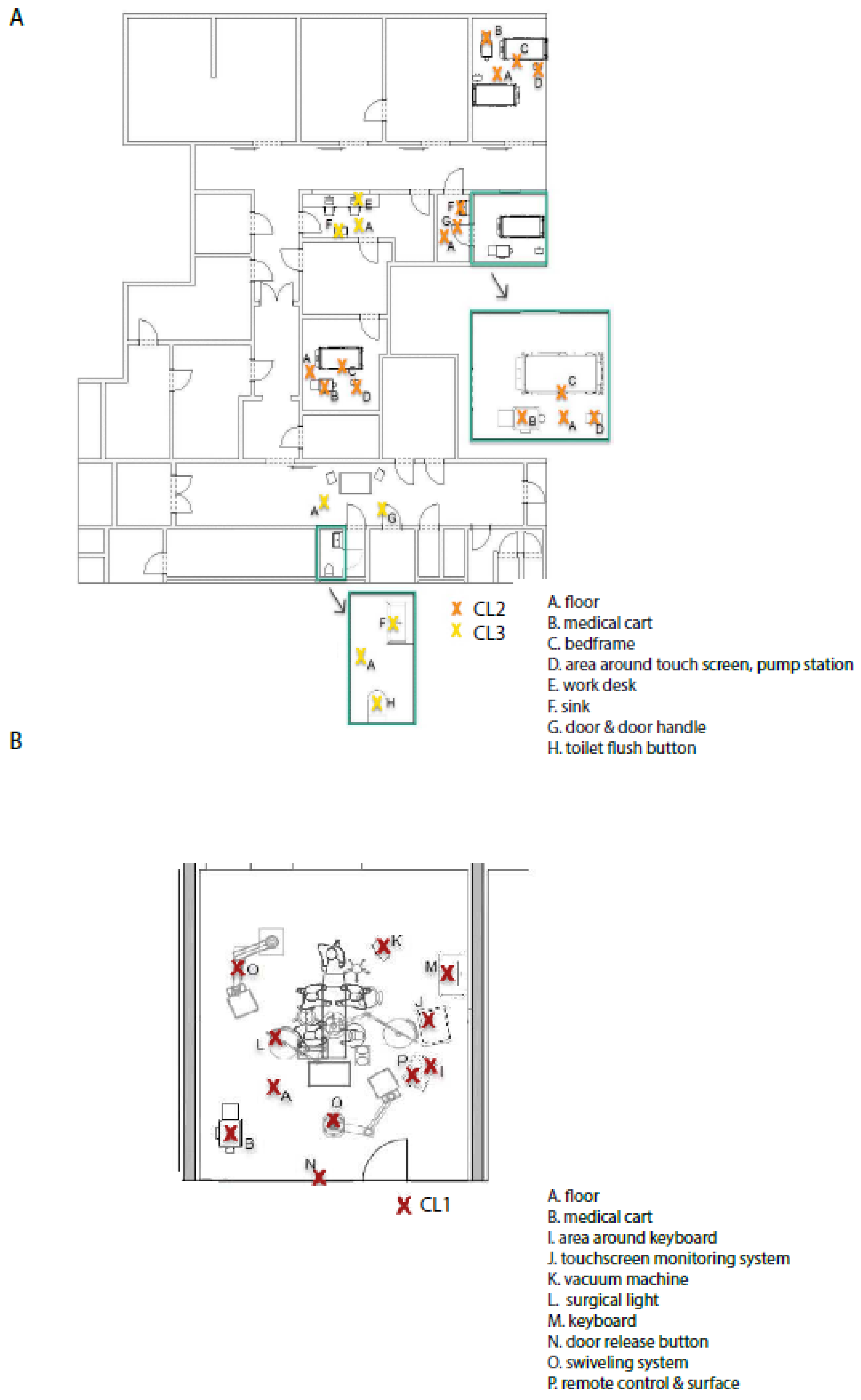

Table S2: Time points and date of sample collection for all three confinement levels (CL).

Department	Time point	Date
**ICU non-patient related areas:**		Level 3 (CL3)
	• t1	2018-03-12
	• t2	2018-04-10
	• t3	2018-05-15
**ICU patient related areas:**		Level 2 (CL2)
	• t1	2018-01-31
	• t2	2018-02-28
	• t3	2018-05-18
**Operating room:**		Level 1 (CL1)
	• t1	2018-03-19
	• t2	2018-04-17
	• t3	2018-05-30

Fig. S2: Flowchart of obtained data and principle data processing workflow.

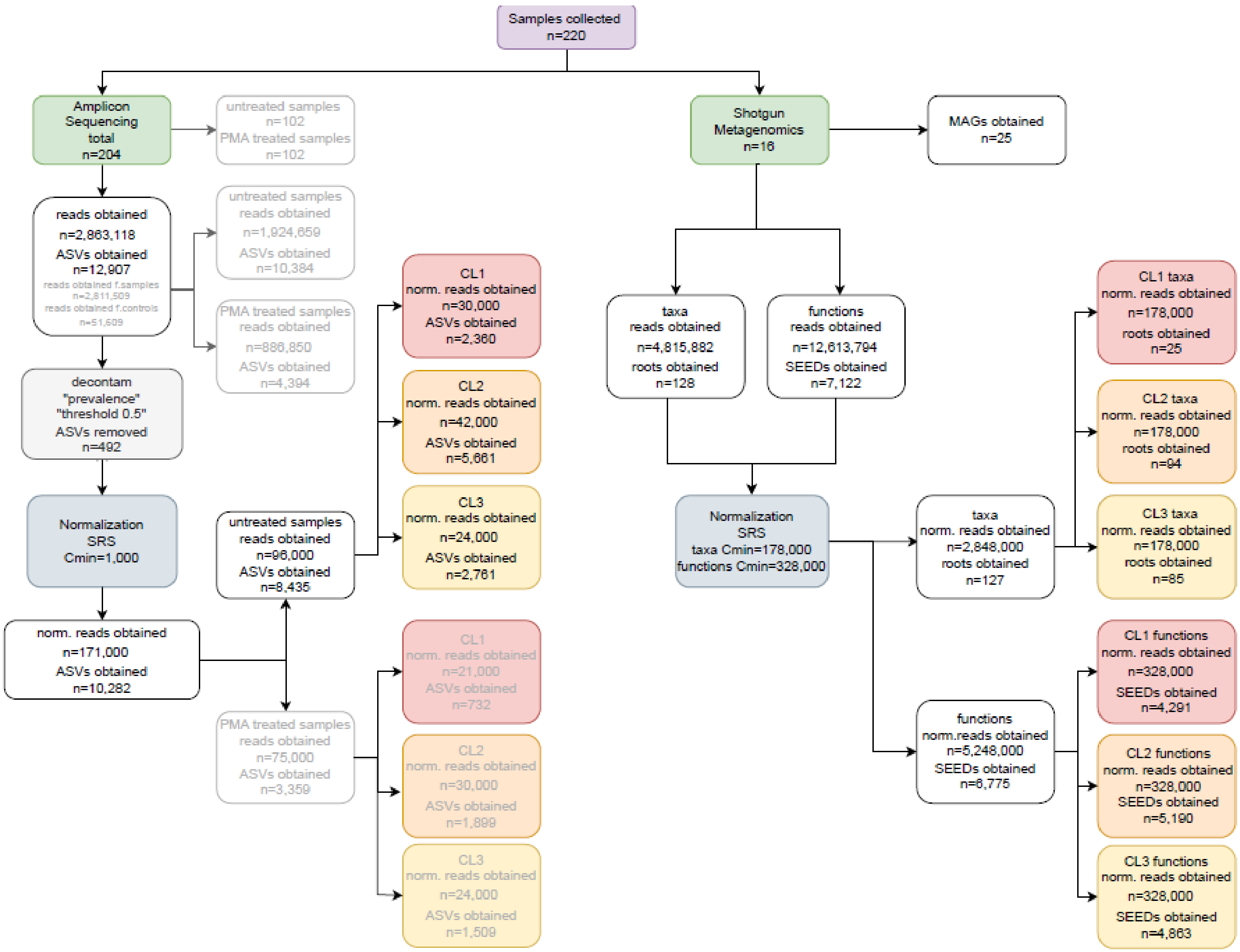

Table S3: Summary of statistical output for alpha and beta diversity of amplicon data

Alpha Diversity Amplicon Data

qiime2 output:

	shannon entropy	observed features	pielou evenness
**CL1**			
MIN	5.13892254	45	0.78843566
MAX	8.18431907	375	0.97582522
AVERAGE	6.64029023	168.933333	0.91847957
SD	0.73038037	80.3732385	0.04512843
**CL2**			
MIN	4.06829477	43	0.70369169
MAX	8.88747511	564	0.97561995
AVERAGE	7.36998948	286.809524	0.92973016
SD	1.07382488	140.918284	0.05771472
**CL3**			
MIN	4.1645694	46	0.75396446
MAX	8.7155701	494	0.97439371
AVERAGE	6.72095281	219.375	0.89464898
SD	1.35268298	133.550776	0.06970333
	all CLs		
SD evenness	0.05859951		
AVERAGE	0.91744406		

MaAsLin 2 alpha diversity amplicon data

feature	metadata	value	coef	stderr	N	N.not.0	pval	qval
shannon entropy	Time point	Time point	0.05747901	0.0202061	96	96	0.0054796	0.015406
shannon entropy	Confinement	CL2	-0.161133554	0.047067	96	96	0.0009248	0.0056688
pielou evenness	Confinement	CL2	-0.200005261	0.0601016	96	96	0.0012597	0.0056688
pielou evenness	Time point	Time point	0.071383019	0.0258019	96	96	0.0068471	0.015406
observed features	Confinement	CL2	0.006194036	0.0026745	96	96	0.0227847	0.0410124
observed features	Time point	Time point	-0.002509132	0.0011482	96	96	0.0314078	0.0471117

MaAsLin 2 beta diversity amplicon data

mean Reference CL 1,2,3

feature	metadata	value	coef	stderr	N	N.not.0	pval	qval
bray.curtis distance	bdiv Time point	bdiv Time point	0.00601202	0.0009955	7710	7710	1.619E-09	1.13E-08
bray.curtis distance	bdiv Confinement	CL3 vs CL1	0.03088405	0.0090432	7710	7710	0.0270937	0.0386344
bray.curtis distance	bdiv Confinement	CL1 vs CL3	0.03088405	0.0090432	7710	7710	0.0270937	0.0386344
bray.curtis distance	bdiv Confinement	CL2 vs CL1	0.03191078	0.0094327	7710	7710	0.0085486	0.0179434
bray.curtis distance	bdiv Confinement	CL1 vs CL2	0.03191078	0.0094327	7710	7710	0.0085486	0.0179434
bray.curtis distance	bdiv Confinement	CL2	0.02700479	0.0087822	7710	7710	0.0173246	0.0297193
bray.curtis distance	bdiv Confinement	CL2 vs CL3	-0.02938788	0.0053121	7710	7710	2.94E-07	5.57E-07
bray.curtis distance	bdiv Confinement	CL3 vs CL2	-0.02938788	0.0053121	7710	7710	2.94E-07	5.57E-07
bray.curtis distance	bdiv Confinement	CL3	-0.03083617	0.0071526	7710	7710	0.0000257	0.0000441
bray.curtis distance	bdiv Confinement	CL1	-0.02317342	0.0104117	7710	7710	0.0346234	0.0479401

Fig S3: Confinement level (CL) prediction by supervised machine learning models based on Random Forest classifications of the amplicon data at genus level and all time points. Relationships between the true positive rate (TPR, on the y-axis) and the false positive rate (FPR, on the x-axis) at various thresholds are illustrated by dashed and solid lines in the ROC plots. Better performance of the sample classification model is indicated by a greater area under the curve (AUC) in comparison to the performance by random chance (diagonal grey line).

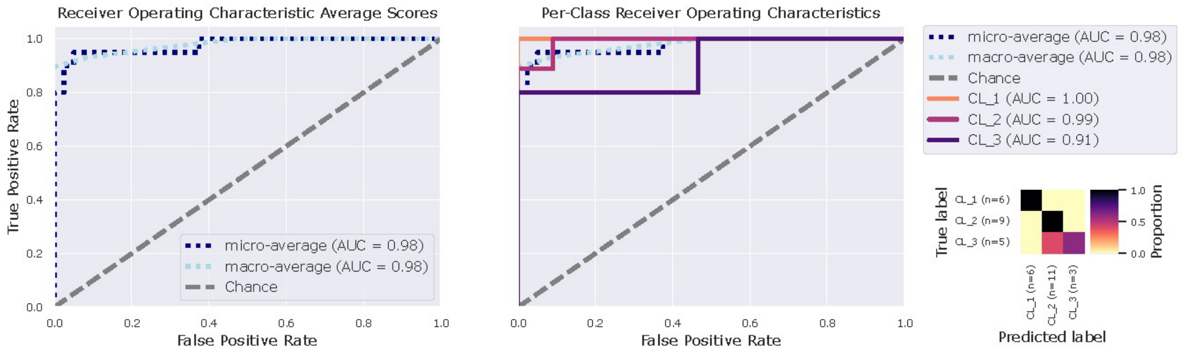

Table S4: Summary of all differential abundance tests performed on the amplicon dataset.

Differential abundance tests Amplicon Data

Statistical Test	Key taxa	statistical output																	
ALDEx 2		CL1 vs CL2																	
		rab.all	rab.win.CL1	rab.win.CL2	diff.btw	diff.win	effect	overlap	we.ep	we.eBH	wi.ep	wi.eBH							
	Achromobacter	1.844564131	7.859024951	0.292821107	-7.404182	4.959943478	-1.32432	0.110383	3.99E-07	0.000123	2.12E-08	7.43E-06							
	Acinetobacter	7.65653356	6.825188862	8.297932092	1.781985	2.908080668	0.538033	0.238791	0.001992	0.093688	0.00011	0.012801							
	Pseudomonas	6.793712265	9.779547676	5.71976332	-4.079495	2.645799326	-1.38739	0.061492	8.13E-10	5.07E-07	2.06E-12	1.72E-09							
	Stenotrophomonas	3.258798938	0.658058252	5.428526286	4.443043	4.287459062	0.927342	0.15869	1.13E-05	0.002434	5.46E-07	0.000139							
		CL1 vs CL3																	
		rab.all	rab.win.CL1	rab.win.CL2	diff.btw	diff.win	effect	overlap	we.ep	we.eBH	wi.ep	wi.eBH							
	Achromobacter	4.009619867	7.779998663	0.772107395	-6.41814	5.353223406	-1.07837	0.13737	1.05E-05	0.001354	5.14E-06	0.000714							
	Acinetobacter	7.602532776	6.740344934	9.932095855	3.104481	3.155786452	0.903461	0.123047	4.81E-06	0.000779	3.78E-07	7.53E-05							
	Pseudomonas	7.827934077	9.65226464	5.516853936	-3.915603	2.934189207	-1.20609	0.078725	8.09E-06	0.000971	1.12E-08	5.60E-06							
	Stenotrophomonas	2.421778472	0.519789835	7.319288418	7.028452	5.172983271	1.226865	0.098243	2.96E-06	0.000438	8.19E-08	2.13E-05							
ANCOM		W	Reject null hypothesis	0	25	50	75	100	0	25	50	75	100	0	25	50	75	100	Percentile
				CL1	CL1	CL1	CL1	CL1	CL2	CL2	CL2	CL2	CL2	CL3	CL3	CL3	CL3	CL3	Group
	Achromobacter	1044	TRUE	1	1	13.5	53.75	526	1	1	1	1	15	1	1	1	1	15	
	Acinetobacter	987	TRUE	1	14.25	29	78.25	643	1	29.75	63	247	859	1	50.25	137	312.25	929	
	Pseudomonas	1044	TRUE	1	45.5	113	295.25	661	1	1	12	20.25	930	1	3.25	11	27.25	423	
	Stenotrophomonas	1045	TRUE	1	1	1	1	128	1	1	5.5	14.25	42	1	6	35.5	142.75	747	
ANCOM2		W	detected_0.9	detected_0.8	detected_0.	detected_0.6													
	Achromobacter	Inf	TRUE	TRUE	TRUE	TRUE													
	Acinetobacter	73	TRUE	TRUE	TRUE	TRUE													
	Pseudomonas	76	TRUE	TRUE	TRUE	TRUE													
	Stenotrophomonas	Inf	TRUE	TRUE	TRUE	TRUE													
ANCOM-BC		Reference CL1																	
		CL2	CL3																
		q-value	q-value																
	Achromobacter	7.68E-17	3.57E-10																
	Acinetobacter	0.003280046	3.69E-08																
	Pseudomonas	1.30E-17	1.13E-10																
	Stenotrophomonas	7.04E-13	1.58E-10																
		Reference CL2																	
		CL1	CL3																
		q-value	q-value																
	Achromobacter	7.68E-17	1																
	Acinetobacter	0.003280046	0.973825894																
	Pseudomonas	1.30E-17	1																
	Stenotrophomonas	7.04E-13	0.184040203																
		Reference CL3																	
		CL1	CL2																
		q-value	q-value																
	Achromobacter	3.57E-10	1																
	Acinetobacter	3.69E-08	0.973825894																
	Pseudomonas	1.13E-10	1																
	Stenotrophomonas	1.58E-10	0.184040203																
MaAsLin2		metadata	value	coef	stderr	N	N.not.0	pval	qval										
	Achromobacter	Confinement	CL1	1.402118814	0.160478	97	30	9.59E-14	6.24E-11										
	Acinetobacter	Confinement	CL1	-0.983785415	0.142816	97	92	6.58E-10	2.14E-07										
	Achromobacter	Time_point	Time_point	0.176972987	0.06033	97	30	0.004222	0.125109										

Fig. S4: Metagenomic comparison of the different confinement levels (CLs). (A and C) Comparison of alpha diversity (Shannon indices) for taxonomy and functions across the different CL (CL1 - red, CL2 - orange, CL3 - yellow). (B and D) Comparison of beta diversity (Bray-Curtis indices) for taxonomy and functions across the different CL (CL1 - red, CL2 - orange, CL3 - yellow). (E-H) Relative abundance of key taxa from the metagenomic data compared between the three CL.

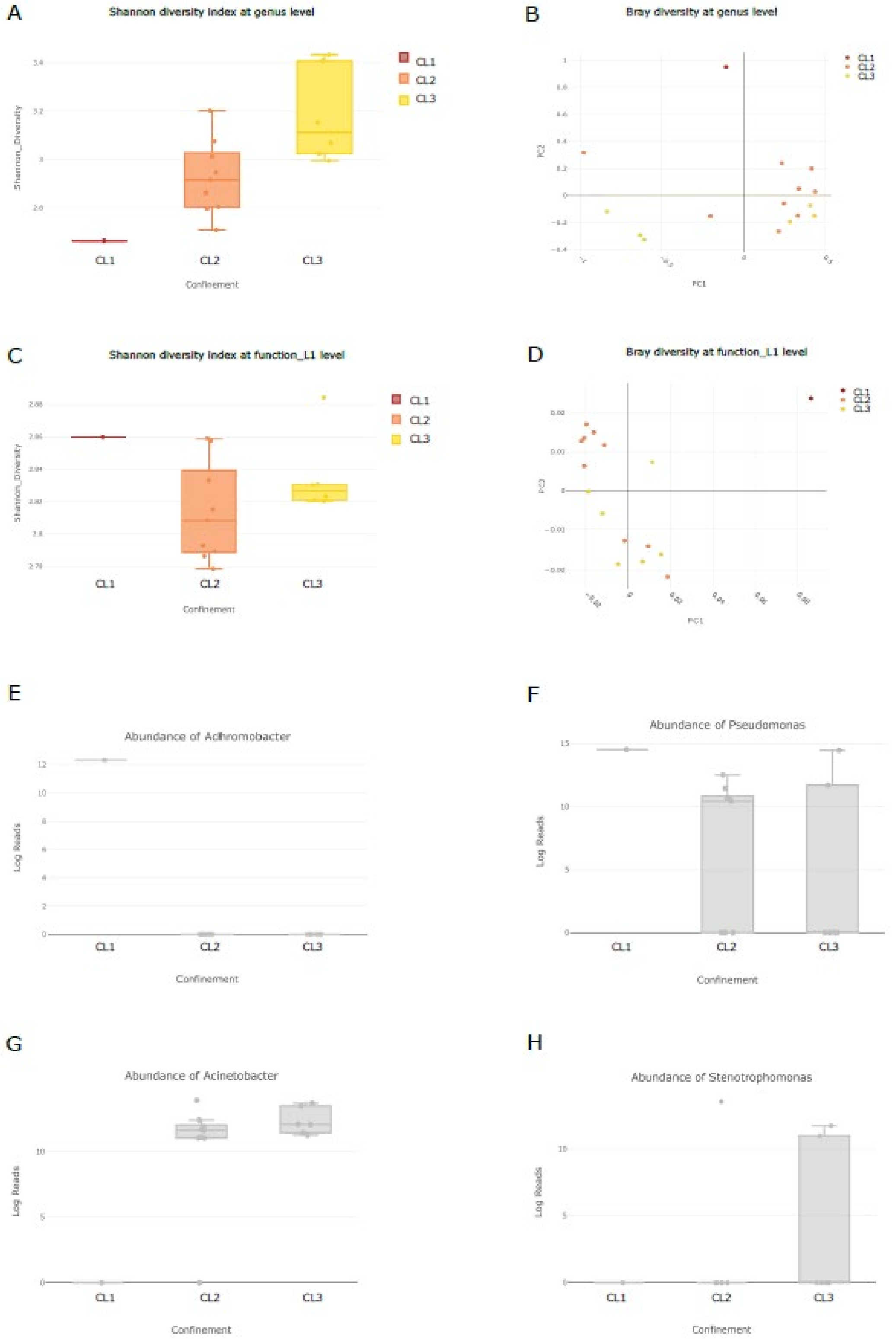

Table S5: Summary of statistical output for taxonomic and functional alpha and beta diversity of the metagenomic data for all three confinement levels**.**

MaAsLin 2

Alpha and beta diversity taxonomy metagenomic data

(Reference CL1)

feature	metadata	value	coef	stderr	N	N.not.0	pval	qval
Bray Curtis	Confinement	CL3	-1.148762556	0.302368799	16	16	0.002947312	0.031482757
shannon	Confinement	CL3	-0.405848791	0.120531627	16	16	0.006283779	0.037171154
Bray Curtis	Confinement	CL2	-0.978836877	0.295837385	16	16	0.006969591	0.037171154
shannon	Confinement	CL2	-0.354108702	0.117928045	16	16	0.012020564	0.045368668

(CL2 vs CL3)

feature	metadata	value	coef	stderr	N	N.not.0	pval	qval
Bray Curtis	Confinement	CL3	-0.169925679	0.144934117	15	15	0.26377287	0.460247926
shannon	Confinement	CL3	-0.051740089	0.053343839	15	15	0.351222985	0.468297313

Alpha and beta diversity functions metagenomic data

(Reference CL1)

feature	metadata	value	coef	stderr	N	N.not.0	pval	qval
Bray Curtis	Confinement	CL2	-1.426450369	0.226794769	16	16	5.92E-05	0.00189527
Bray Curtis	Confinement	CL3	-1.272082358	0.23180188	16	16	0.000189692	0.002023382
shannon	Confinement	CL2	-0.329399308	0.06353909	16	16	0.000301779	0.002414232
shannon	Confinement	CL3	-0.19768763	0.064941888	16	16	0.011165138	0.04123194

(CL2 vs CL3)

feature	metadata	value	coef	stderr	N	N.not.0	pval	qval
shannon	Confinement	CL3	0.131711678	0.029948405	15	15	0.000868504	0.003691033
Bray Curtis	Confinement	CL3	0.154368011	0.107696026	15	15	0.177289958	0.257876302

Fig. S5: Growth values (GRiD) of obtained metagenome assembled genomes (MAGs). (A) Boxplots of GRiD values of all obtained MAGs. (B) Boxplots for GRiD value comparison between the different MAGs obtained from the different confinement levels.

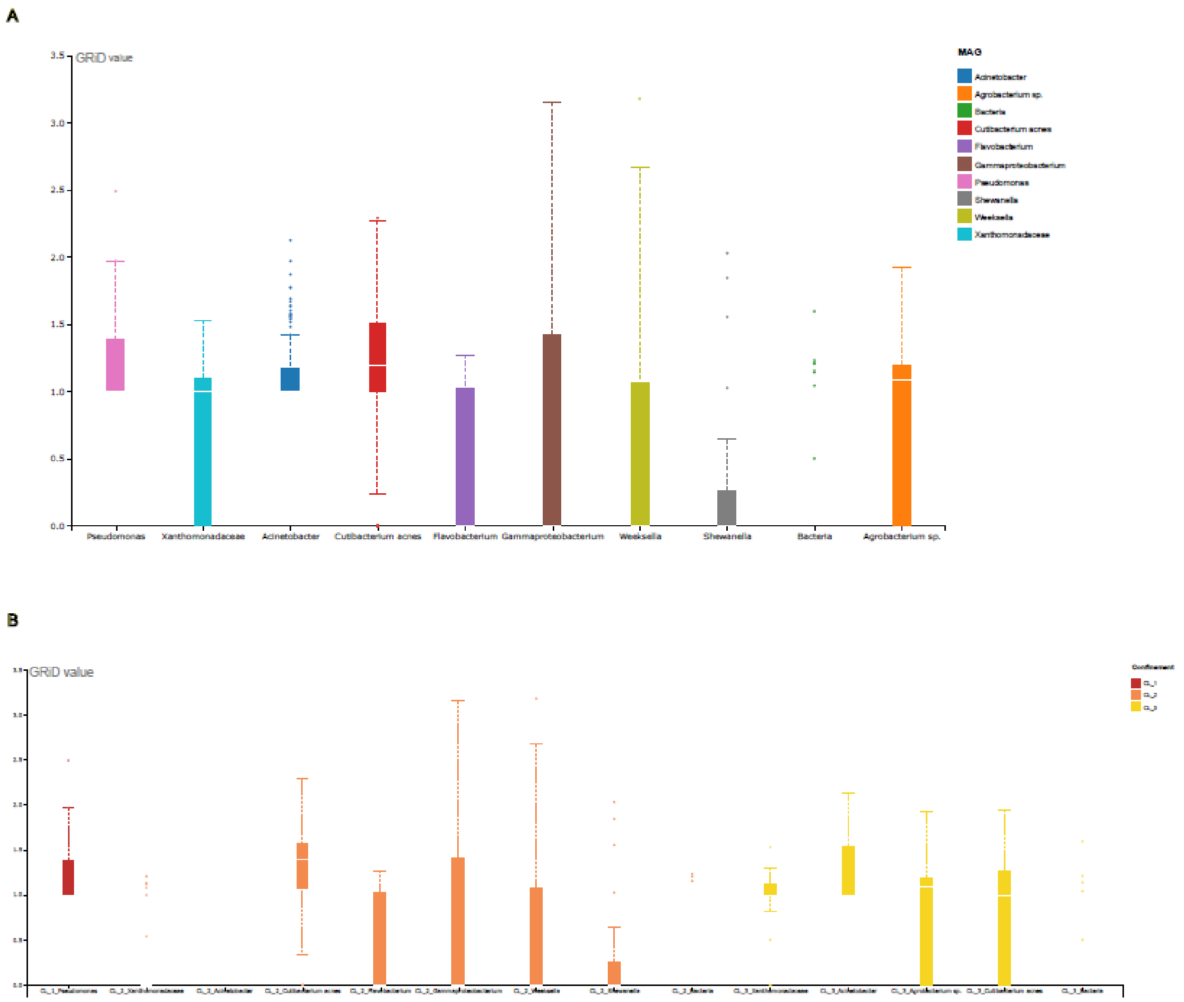

Supplement: Supplemental material — Supplemental tables and figures. [file msystems.00726-24-s0001.pdf]
